# MGMT inactivation as a new biomarker in patients with advanced biliary tract cancers

**DOI:** 10.1002/1878-0261.13256

**Published:** 2022-06-13

**Authors:** Monica Niger, Federico Nichetti, Andrea Casadei‐Gardini, Federica Morano, Chiara Pircher, Elena Tamborini, Federica Perrone, Matteo Canale, Daniel B. Lipka, Andrea Vingiani, Luca Agnelli, Anna Dobberkau, Jennifer Hüllein, Felix Korell, Christoph E. Heilig, Sara Pusceddu, Francesca Corti, Michele Droz, Paola Ulivi, Michele Prisciandaro, Maria Antista, Marta Bini, Laura Cattaneo, Massimo Milione, Hanno Glimm, Bruno C. Köhler, Giancarlo Pruneri, Daniel Hübschmann, Stefan Fröhling, Vincenzo Mazzaferro, Filippo Pietrantonio, Maria Di Bartolomeo, Filippo de Braud

**Affiliations:** ^1^ Medical Oncology Department Fondazione IRCCS Istituto Nazionale dei Tumori di Milano Italy; ^2^ Computational Oncology Group, Molecular Precision Oncology Program National Center for Tumor Diseases (NCT) and German Cancer Research Center (DKFZ) Heidelberg Germany; ^3^ Vita‐Salute San Raffaele University Milan Italy; ^4^ Department of Medical Oncology San Raffaele Scientific Institute IRCCS Milan Italy; ^5^ Pathology and Laboratory Medicine Fondazione IRCCS Istituto Nazionale dei Tumori di Milano Italy; ^6^ Biosciences Laboratory IRCCS Istituto Romagnolo per lo Studio dei Tumori “Dino Amadori” (IRST) Meldola Italy; ^7^ Section Translational Cancer Epigenomics, Division of Translational Medical Oncology German Cancer Research Center (DKFZ) & National Center for Tumor Diseases (NCT) Heidelberg Germany; ^8^ Heidelberg and Partner Sites German Cancer Consortium (DKTK) Heidelberg Germany; ^9^ Department of Hematology & Oncology University Hospital Heidelberg Germany; ^10^ Division of Translational Medical Oncology German Cancer Research Center (DKFZ) & National Center for Tumor Diseases (NCT) Heidelberg Germany; ^11^ Division of HPB, General Surgery and Liver Transplantation, Department of Surgery Fondazione IRCCS Istituto Nazionale Tumori di Milano Italy; ^12^ Department of Oncology and Hemato‐Oncology University of Milan Italy; ^13^ Department of Translational Medical Oncology National Center for Tumor Diseases (NCT) Dresden and German Cancer Research Center (DKFZ) Germany; ^14^ Center for Personalized Oncology National Center for Tumor Diseases (NCT) Dresden and University Hospital Carl Gustav Carus Dresden at TU Dresden Germany; ^15^ Department of Medical Oncology, University Hospital Heidelberg National Center for Tumor Diseases (NCT) Heidelberg Germany; ^16^ Liver Cancer Center Heidelberg University Hospital Heidelberg Germany; ^17^ Heidelberg Institute for Stem Cell Technology and Experimental Medicine Germany

**Keywords:** biliary tract cancer, biomarker, cholangiocarcinoma, MGMT, molecular profiling, temozolomide

## Abstract

Biliary tract cancers (BTCs) have poor prognosis and limited therapeutic options. The impact of *O* ^6^‐methylguanine‐DNA methyltransferase (*MGMT*) inactivation in advanced BTC patients is not established. We investigated the prevalence, prognostic, and predictive impact of *MGMT* inactivation in two multicenter cohorts. MGMT inactivation was assessed through PCR and immunohistochemistry (IHC) in an Italian cohort; the results were then externally validated using RNA sequencing (RNA‐seq) data from the BTC subcohort of the Molecularly Aided Stratification for Tumor Eradication Research (MASTER) precision oncology program of the National Center for Tumor Diseases Heidelberg and the German Cancer Consortium. Among 164 Italian cases, 18% presented *MGMT* promoter hypermethylation (> 14%) and 73% had negative MGMT protein expression. Both were associated with worse overall survival (OS; HR 2.31; *P* < 0.001 and HR 1.99, *P* = 0.012, respectively). In the MASTER cohort, patients with lower *MGMT* mRNA expression showed significantly poorer OS (median OS [mOS] 20.4 vs 31.7 months, unadjusted HR 1.89; *P* = 0.043). Our results suggest that *MGMT* inactivation is a frequent epigenetic alteration in BTC, with a significant prognostic impact, and provide the rationale to explore DNA‐damaging agents in *MGMT*‐inactivated BTCs.

## Graphical abstract

The impact of *MGMT* inactivation in patients with advanced biliary tract cancer (BTC) is not established. We investigated its prevalence, prognostic, and predictive impact, assessed through methylation‐specific PCR and IHC, in a large Italian cohort. Then we evaluated *MGMT* inactivation through RNA‐seq and DNA methylation analysis in an independent German cohort. Overall, *MGMT* inactivation or reduced expression consistently resulted in poorer survival.

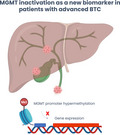

**Abbreviations**
5‐FU: 5‐fluorouracil
1L: first‐line
BTCs: biliary tract cancers
CI: confidence interval
dCCA: distal cholangiocarcinoma
DKTK: German Cancer Consortium
ECOG PS: Eastern Cooperative Oncology Group performance status
GBC: Gall bladder cancer
HR: hazard ratio
iCCA: intrahepatic cholangiocarcinoma
IHC: immunohistochemistry
INT: Fondazione IRCCS Istituto Nazionale Tumori Of Milan
IQR: interquartile range
MASTER: Molecularly Aided Stratification For Tumor Eradication Research
MGMT: O^6^‐methylguanine DNA methyltransferase
NA: not available
NCT: Nationale Centrum Für Tumorerkrankungen
OS: overall survival
pCCA: perihilar cholangiocarcinoma
PD: progressive disease
PFS: progression‐free survival
RNA‐seq: RNA sequencing
TMZ: temozolomide
TPM: transcripts per kilobase million
VIMP: variable importance

## 1 Introduction

Biliary Tract Cancer (BTC) is a rare disease with overall poor prognosis and limited therapeutic options [1, 2]. Emerging evidence reveals that BTC is heterogeneous from a pathological and molecular perspective, with significant differences between intrahepatic cholangioarcinoma (iCCA), extrahepatic cholangiocarcinoma (eCCA), and gallbladder cancer (GBC) [3, 4, 5, 6, 7]. There is a growing evidence in favor of biomarker‐directed treatments in BTC, such as pemigatinib and infigratinib for CCA with *FGFR2* fusions [8, 9], ivosidenib for *IDH1* mutated CCAs [10], and *BRAF* plus *MEK* inhibitors in *BRAF* V600E mutated or *HER2* inhibitors in *ERRB2* amplified/mutated BTCs [11, 12, 13]. Finally, there is an increasing amount of data regarding the identification of DNA damage repair aberrations and distinct DNA hypermethylation patterns in these cancers [3, 4, 14].

O^6^‐methylguanine‐DNA methyltransferase (*MGMT*) encodes for a key DNA repair enzyme, responsible for the elimination of alkyl groups from the O6‐position of guanine. *MGMT* promoter methylation, leading to reduction of MGMT expression, ultimately results in diminished DNA‐repair of O6‐alkylguanine adducts and enhanced sensitivity to alkylating agents, such as temozolomide (TMZ) [15]. *MGMT* promoter hypermethylation is a validated biomarker for the efficacy of TMZ in glioblastoma [15]. Similarly, both *MGMT* promoter hypermethylation and reduced/absent MGMT expression are described in a variety of gastrointestinal malignancies, including colorectal cancers [16, 17]. In patients with metastatic colorectal cancer, several trials showed that *MGMT* silencing is a potential biomarker to select patients for TMZ‐based treatment [18, 19, 20].

In the present study, we investigate the prevalence, as well as the prognostic and predictive impact of *MGMT* inactivation in two independent series of advanced BTC cases. Moreover, we report the first evidence on the activity of TMZ in patients with BTC.

## 2 Materials and methods

### 2.1 Patient population and study objectives

We first conducted a multicenter observational study at Fondazione IRCCS Istituto Nazionale Tumori of Milan (INT) and Istituto Scientifico Romagnolo per lo Studio e la Cura dei Tumori (IRST) IRCCS, Meldola. From October 2017 to November 2020, we included all patients fulfilling the following eligibility criteria: (a) histologically/cytologically confirmed diagnosis of BTC; (b) unresectable primary tumor and/or evidence of metastases; and (c) available archival tumor tissue for molecular profiling.

Baseline demographic, clinical, and biological data were collected through an electronic database. All patients were followed up until death, loss to follow up or data cut‐off date (December 10, 2020).

The primary aim was to investigate the frequency and percentage of *MGMT* promoter hypermethylation in advanced BTC patients. Secondary aims were: (a) to investigate the prognostic impact of *MGMT* promoter hypermethylation in terms of overall survival (OS); (b) to investigate the predictive role of *MGMT* promoter hypermethylation in first‐line (1L) of standard chemotherapy in terms of 1L‐progression‐free survival (PFS); (c) to explore the potential effect on 1L‐PFS of the interaction between *MGMT* promoter hypermethylation and use of platinum‐based chemotherapy; (d) to evaluate the prognostic and predictive role of MGMT expression as evaluated by immunohistochemistry (IHC), given the growing evidence of the role of IHC as complementary assessment tool of MGMT status [18, 19, 21]; (e) to explore the association between *MGMT* promoter hypermethylation and the tumor molecular profile; and (f) to report safety and efficacy data of a case series of patients treated with TMZ‐related regimens.

The study was approved by the Institutional Review Boards of the two institutions and was conducted in accordance with the Declaration of Helsinki; patients provided written informed consent.

### 2.2 External validation cohort

To externally validate the proof‐of‐concept results of the Italian cohorts with different omics layers, data from the BTC subcohort of the Molecularly Aided Stratification for Tumor Eradication Research (MASTER) study conducted by NCT Heidelberg and the German Cancer Consortium (DKTK) were interrogated [22]. NCT/DKTK MASTER is a registry trial and analytical platform for prospective, multi‐omics‐guided stratification of patients with advanced cancers diagnosed at a young age (< 51 years) or with rare cancers, including BTC, and comprises broad molecular profiling including RNA sequencing (RNA‐seq) and DNA methylation analysis. For the analyses presented here, all patients enrolled in MASTER from March 2012 to February 2021 with histologically/cytologically confirmed diagnosis of BTC and available clinical and RNA‐seq data were considered. The study was approved by the Ethics Committee of the Medical Faculty of Heidelberg University and was conducted in accordance with the Declaration of Helsinki; patients provided written informed consent.

### 2.3 MGMT status assessment

For samples from the two Italian centers, hematoxylin–eosin slides were reviewed by an expert pathologist to select an area comprising at least 50% tumor cells. DNA was extracted as previously described [20]. *MGMT* promoter methylation was assessed by MGMT plus® Diatech Pharmacogenetics (Jesi, Italy), which analyzes 10 CpG islands spanning the promoter region (chr10:131, 256, 507–131, 256 556). Briefly, after bisulfite conversion of the extracted DNA (range: 200–500 ng) and its amplification by using primers specific for methylated and unmethylated DNA sequences, a pyrosequencing of the obtained templates was performed. The final result provided the number of methylated CpG islands present in the promoter region of the *MGMT* gene, expressed as a percentage. When sufficient residual tumor tissue was available, MGMT expression was assessed by immunohistochemistry (IHC), as previously described [19]. Additional tumor molecular characterization was non‐uniformly performed through the Ion Torrent Personal Genome platform (50 genes ‘Hotspot Cancer Panel, Ion Torrent®’; Life Technologies®, Waltham, MA, USA), as in [23], or the FoundationOne®CDx panel [24].

In the Molecularly Aided Stratification For Tumor Eradication Research on Biliary Tract Cancers (MASTER BTC) cohort, processing of tumor specimens and technical details of RNA‐seq analyses are described in the study done by Horak et al. [22]. *MGMT* mRNA expression was measured as transcripts per kilobase million (TPM). The methylation status of the MGMT promoter derived from available Illumina Infinium EPIC array data (*n* = 50) using the MGMT‐STP27 prediction model [25] as implemented in the mgmtstp27 r package (https://github.com/badozor/mgmtstp27).

### 2.4 Statistical methods

*MGMT* promoter methylation was first analyzed as a continuous variable (i.e. as number of methylated CpGs present in the promoter region of the *MGMT* gene, expressed as a percentage), with non‐linear effects assessed by means of restricted cubic splines. Subsequently, the optimal cutoff for OS prediction was calculated using the maximally selected rank statistic, as described by Hothorn and Lausen [26]. The cutoff was then adopted also in 1L‐PFS analyses for consistency. Cases from the MASTER BTC validation cohort were grouped according to MGMT mRNA expression (TPM value), using the median value as cutoff. The Fisher's exact test, Chi‐squared test, and Wilcoxon–Mann–Whitney test were used to study the distribution of categorical and continuous variables, respectively, according to dichotomized *MGMT* status, as appropriate. Cohen's kappa was used to measure the agreement of PCR and IHC assays assessing *MGMT* status.

Median follow‐up was quantified with the reverse Kaplan–Meier estimator [27]. Survival analysis methods were used to analyze OS and 1L‐PFS. OS was calculated from the date of advanced disease diagnosis to death or last follow‐up, while 1L‐PFS was calculated from the date of first‐line treatment start to the first event [i.e. progressive disease (PD, as defined as according to RECIST v1.1) or death]. Patients who had not undergone PD or death at the time of data cut‐off were censored at their last disease evaluation. Survival curves and related descriptive statistics were obtained with the Kaplan–Meier method and comparisons between curves were performed with the logrank test. Multivariate analyses in the Italian cohorts were performed with a two‐step strategy: at first, covariates were modeled with a random forest method [28] according to the following endpoints: (a) imputation of missing data at random, using adaptive tree imputation; (b) selection of relevant covariates, by taking the top‐ranked variables by matching the variable importance (VIMP) and Minimal Depth statistics. Then, multivariable Cox proportional models were designed using the selected variables and imputed missing data, and results were summarized using hazard ratios (HRs), together with the corresponding 95% confidence intervals (CI). Interaction terms were used to investigate the interplay between *MGMT* methylation status and use of platinum‐based first‐line chemotherapy in terms of PFS.

In the MASTER BTC cohort, OS was calculated from the date of first disease diagnosis, and available data on patients' age, gender, and primary tumor location, prior tumor resection and use of adjuvant therapy were used as covariates in multivariate Cox proportional models.

A threshold of significance of 0.05 was set for all statistical evaluations. Statistical analyses were performed in the r (Version 4.0.3) and rstudio (Version 1.3.1073) software [R Foundation for Statistical Computing, Vienna, Austria].

## 3 Results

### 3.1 Italian cohort

#### 3.1.1 Patient population

Of a total number of 230 patients, 164 cases were successfully profiled for *MGMT* promoter methylation status (patients' flow depicted in Fig. S1). Baseline characteristics are displayed in Table 1.

**Table 1:** Patients' characteristics in the Italian study cohort. 5‐FU, 5‐fluorouracil; dCCA, distal cholangiocarcinoma; ECOG PS, Eastern Cooperative Oncology Group Performance Status; iCCA, intrahepatic cholangiocarcinoma; IHC, immunohistochemistry; IQR, interquartile range; MGMT, O^6^‐methylguanine DNA methyltransferase; NA, not available; pCCA, perihilar cholangiocarcinoma.

Characteristic	*N* (%)
Total number of patients	164 (100.0)
Age in years [median (IQR)]	66 (57–72)
Gender	
Male	71 (43.3)
Female	93 (56.7)
ECOG PS	
0–1	128 (85.3)
≥ 2	22 (14.7)
NA	14
Primary tumor location	
iCCA	100 (61.0)
pCCA	7 (4.3)
dCCA	23 (14.0)
Gall bladder	34 (20.7)
Primary tumor resected	
Yes	107 (65.2)
No	57 (34.8)
Adjuvant treatment	
Yes	46 (28.0)
No	61 (37.2)
Not applicable	57 (34.8)
Diagnosis of advanced disease^a^	
Synchronous	85 (51.8)
Metachronous	79 (48.2)
Liver‐limited disease	48 (29.3)
Sites of metastatic disease	
Lymph nodes	78 (47.6)
Bones	11 (6.7)
Liver	90 (54.9)
Lungs	34 (20.7)
Peritoneum	31 (18.9)
Total lines of treatment for unresectable/metastatic disease	
Best supportive care	4 (2.4)
1	43 (26.2)
2	40 (24.4)
> 2	42 (25.6)
NA	35
First line treatment regimen	
Best Supportive Care	4 (2.4)
Capecitabine/5‐FU	12 (7.3)
Gemcitabine	12 (7.3)
Capecitabine/5‐FU + Oxaliplatin	15 (9.1)
Gemcitabine + Oxaliplatin	11 (6.7)
Gemcitabine + Cisplatin	88 (53.7)
Other	18 (11.0)
NA	4
Platinum‐based first line regimen^b^	
Yes	115 (73.7)
No	41 (26.3)
NA	8
*MGMT* promoter methylation [median (IQR)]	5 (3–10)
MGMT expression by IHC	
Positive	27 (27.0)
Weakly positive	32 (32.9)
Negative	41 (41.0)
NA	64

^a^ Diagnosis of advanced disease was considered synchronous if occurring < 6 months from primary tumor detection, metachronous if ≥ 6 months.

^b^ One patient was treated with Carboplatin‐based chemotherapy.

At data cut‐off date, 138 patients had experienced disease progression on first‐line treatment, and 132 patients had died. The median follow‐up was 58.0 months [interquartile range (IQR): 34.9–79.9], with a median 1L‐PFS of 4.9 months (IQR: 2.7–9.1) and a median OS of 17.3 months (IQR: 8.0–36.1). First‐line‐PFS was longer in patients treated with 1‐line platinum‐based chemotherapy (5.4 vs 3.5 months, *P* = 0.05), while mOS did not differ according to use of platinum (19.8 vs 10.7, *P* = 0.2).

#### 3.1.2 Prognostic and predictive impact of *MGMT* promoter hypermethylation

We first investigated the impact of *MGMT* promoter methylation, as a continuous variable, on OS. In a univariate Cox regression model, higher *MGMT* promoter methylation values were associated with an increased risk of death (unadjusted HR 1.02 per 1% increase in methylation value, 95% CI 1.01–1.04, *P* = 0.009, Fig. S2).

Then, we identified 14% as the best percentage cutoff of *MGMT* promoter methylation for the prediction of OS. Overall, 135 (82%) and 29 (18%) patients were thus aggregated in the low (≤ 14%) and high (> 14%) *MGMT* promoter methylation groups, respectively. According to each tumor entity, high *MGMT* hypermethylation was observed in 10 (10%) iCCA, 6 (26%) dCCA, and 13 (38%) of GBC patients.

Patients and disease characteristics according to *MGMT* promoter methylation are summarized in Table S1. Of note, highly methylated cases more frequently had worse baseline Eastern Cooperative Oncology Group Performance Status [ECOG PS ≥ 2 in 11/29 (39%) vs 11/135 (9%), respectively] and did not receive first‐line platinum‐based chemotherapy [16/29 (57%) vs 25/135 (19%)].

**Fig. 1:**
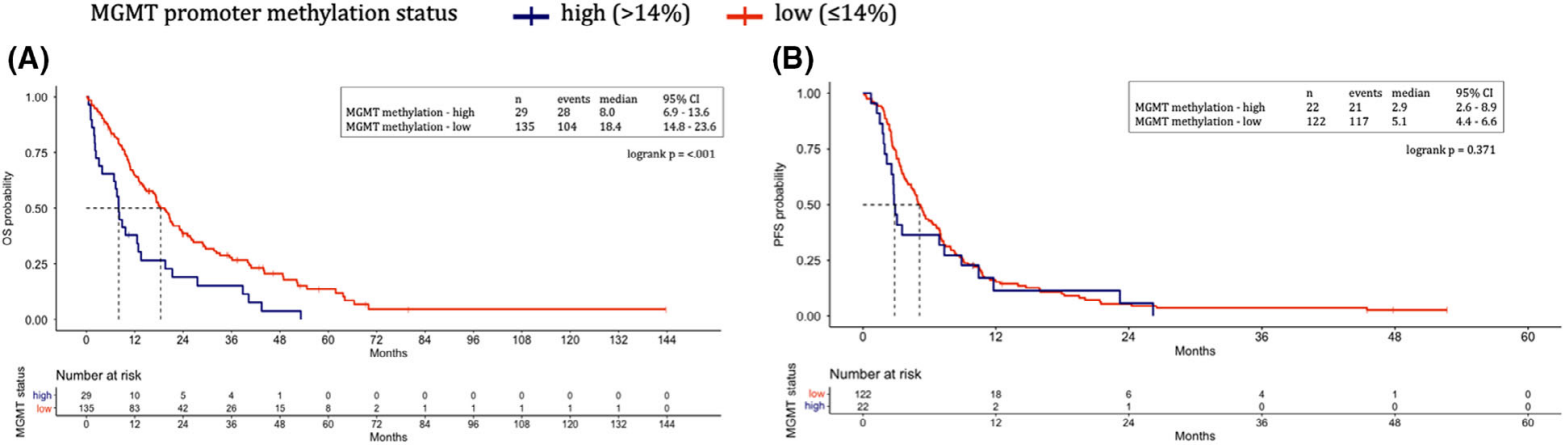
*MGMT* promoter methylation status. Overall survival (A) and first‐line progression‐free survival (B) represented through Kaplan–Meier curves according to *MGMT* promoter methylation status in the Italian cohort. Patients with *MGMT* hypermethylation (blue) experienced worse median overall survival as compared with patients with low *MGMT* methylation (red) (A). *MGMT* promoter methylation values did not impact the progression‐free survival of patients treated with first‐line systemic treatment for advanced disease (B). Dotted lines indicate the median survival time. MGMT, *O* ^6^‐methylguanine‐DNA methyltransferase. [Colour figure can be viewed at wileyonlinelibrary.com]

Patients with *MGMT* hypermethylation experienced worse median OS (mOS 8.0 vs 18.4 months, unadjusted HR 2.16; 95% CI 1.41–3.29; *P* < 0.001, Fig. 1A).

Then, we investigated the impact of *MGMT* promoter methylation on patient OS, as evaluated as a dichotomous variable with the 14% cutoff, in a multivariable model. Via a Random Survival Forest approach, the following covariates were selected as the most relevant, together with *MGMT* status (i.e. variables in the lower left quadrant of Fig. S3): ECOG PS, patient age, previous adjuvant treatment, presence of bone and lung metastases, and number of treatment lines for advanced disease. In a multivariable Cox model including these variables, *MGMT* status was confirmed as an independent negative prognostic factor for OS (adjusted HR 2.31; 95% CI 1.44–3.71; *P* < 0.001, Table 2).

**Table 2:** Multivariable cox proportional hazards model for overall survival. The HR for continuous variables is expressed as the HR variation per unit increase of the variable value (i.e. per 1 year increase). CI, confidence interval; ECOG PS, Eastern Cooperative Oncology Group Performance Status; HR, Hazard Ratio; MGMT, O^6^‐methylguanine DNA methyltransferase.

Variables		HR	95% CI	*P*
*MGMT* promoter methylation	High (> 14%) vs low (≤ 14%)	2.31	1.44–3.71	< 0.001
ECOG PS	≥ 2 vs 0–1	1.68	1.01–2.79	0.044
Age	Continuous	1.03	1.01–1.05	< 0.001
Adjuvant treatment	Not applicable (no tumor resection) vs no	1.51	0.99–2.30	0.054
	Yes vs no	1.51	0.96–2.40	0.071
Lines of treatment for unresectable/metastatic disease	1 vs best supportive care	1.12	0.32–3.88	0.860
	2 vs best supportive care	0.82	0.24–2.79	0.755
	> 2 vs best supportive care	0.70	0.20–2.40	0.570
Presence of lung metastases	Yes vs No	1.43	0.93–2.19	0.101
Presence of bone metastases	Yes vs No	2.00	1.01–3.95	0.045

Concerning 1L‐PFS, outcome data were available for 144 (88%) patients. *MGMT* promoter methylation values were not significantly associated with 1L‐PFS, neither as a continuous variable (unadjusted HR 1.01 per 1% increase in methylation value, 95% CI 0.99–1.03, *P* = 0.219, Fig. S4), nor as dichotomized variable (mPFS 2.9 vs 5.1 months, HR 1.24, 95% CI 0.78–1.98, *P* = 0.363, Fig. 1B).

Next, the potential interaction between *MGMT* promoter hypermethylation and use of platinum‐based chemotherapy as first‐line regimen was assessed. In a multivariate Cox model including the most relevant covariates affecting PFS (i.e. the use of platinum‐based chemotherapy, patient age, previous adjuvant treatment, and presence of lung metastases; Fig. S5), a significant interaction between these two factors was observed (*P* for interaction = 0.038, Table S2).

First‐line‐PFS Kaplan–Meier curves of patients with high (> 14%) and low (≤ 14%) *MGMT* promoter methylation, stratified according to the use of platinum‐based regimens as first‐line chemotherapy, are shown in Fig. S6a,b: patients in the high *MGMT* methylation subgroup treated with first‐line platinum‐based chemotherapy showed longer 1L‐PFS compared to those treated with platinum‐free regimens (mPFS 8.1 vs 2.8 months, unadjusted HR 0.28; 95% CI 0.09–0.85; *P* = 0.018), while no difference was highlighted in the low *MGMT* methylation subgroup (mPFS 5.4 vs 4.5 months, unadjusted HR 0.86; 95% CI 0.55–1.36; *P* = 0.530). As an alternative representation of the interaction, the impact of MGMT promoter methylation on 1L‐PFS was assessed according to the chemotherapy regimen: in patients not treated with platinum salts, patients with high MGMT promoter methylation had significantly poorer 1L‐PFS (mPFS 2.8 vs 4.5 months, unadjusted HR 3.95; 95% CI 1.69–9.25; *P* < 0.001), while this effect was neutralized in patients treated with platinum‐based chemotherapy (mPFS 8.1 vs 4.5 months, unadjusted HR 0.83; 95% CI 0.44–1.55; *P* = 0.550).

#### 3.1.3 Exploratory analysis of MGMT expression as a biomarker

Within the study cohort, 100 (61%) patients had evaluable tumor tissue for MGMT expression by IHC. In detail, MGMT expression was positive in 27 (27%), weakly positive in 32 (32%), and negative in 41 (41%) patients. The impact of MGMT expression on patient OS and PFS was first explored in the three‐level definition, showing that weakly positive and negative cases had similar outcomes (see Fig. S7a,b). For further analyses, these two groups were thus merged and considered as negative.

*MGMT* methylation and IHC expression had poor agreement (Cohen's κ −0.003, 95%CI ‐0.068–0.061). Patient and disease characteristics according to MGMT expression by IHC are summarized in Table S3.

**Fig. 2:**
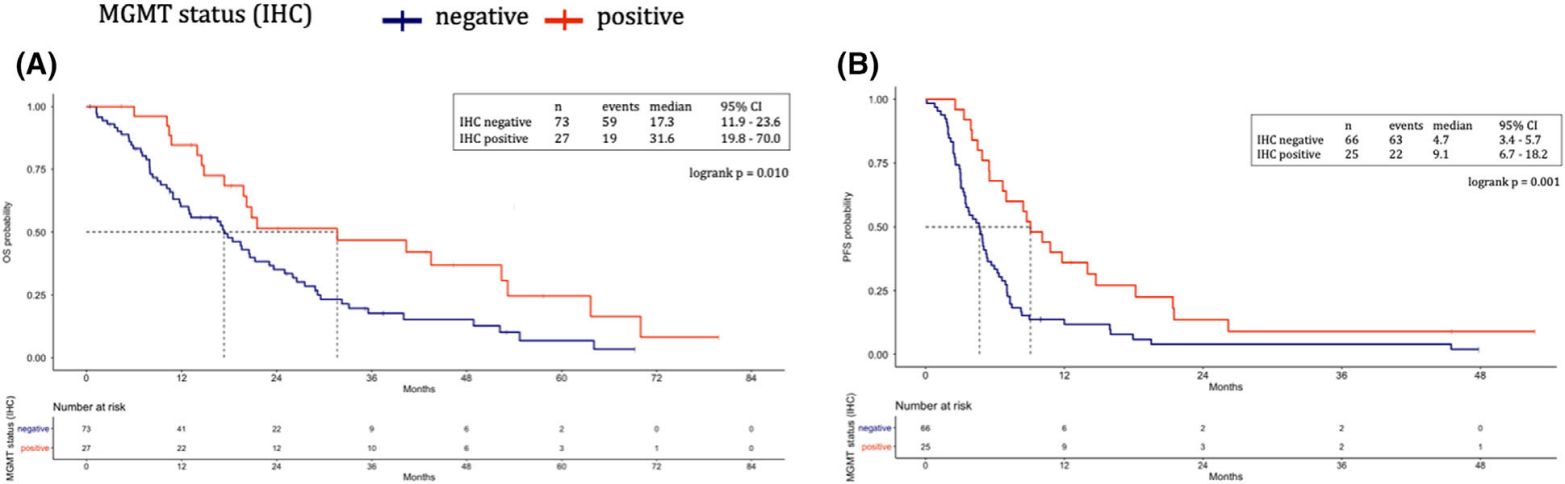
MGMT expression assessed by immunohistochemistry. Overall survival (A) and first‐line progression‐free survival (B) represented through Kaplan–Meier curves according to MGMT status assessed by IHC in the Italian cohort. Patients with negative MGMT expression (blue) showed significantly worse overall survival (A) and progression‐free survival with first‐line systemic treatment (B), as compared with patients with positive MGMT expression (red). Dotted lines indicate the median survival time. MGMT, *O* ^6^‐methylguanine‐DNA methyltransferase. [Colour figure can be viewed at wileyonlinelibrary.com]

Patients with negative MGMT expression showed significantly worse OS (mOS 17.3 vs 31.6 months, unadjusted HR 1.99; 95% CI 1.17–3.41; *P* = 0.012) and 1L‐PFS (mPFS 4.7 vs 9.1 months, unadjusted HR 2.24; 95% CI 1.36–3.68; *P* = 0.001; Fig. 2A,B). No significant interaction with use of platinum‐based chemotherapy regimens was observed (*P* for interaction = 0.898).

#### 3.1.4 Molecular profiling

Among the study cohort, 130 (79%) cases treated at INT underwent additional tumor molecular profiling, as depicted in Fig. S8. The most frequent alterations identified were *TP53* mutations (29%), *KRAS* mutations (18%), followed by *FGFR2* alterations (11% and 4% had *FGFR2* fusions and mutations, respectively), *IDH1* mutations (10%), and *PIK3CA* mutations (9%). Among cases in the *MGMT* promoter hypermethylation subgroup, only 9/29 (31%) were profiled. In this group, no cases harbored *IDH1/2* mutations, while 3/9 (33%) were found as *FGFR2* rearranged.

### 3.2 MASTER BTC cohort

#### 3.2.1 External validation of the prognostic role of MGMT

We analyzed 76 BTC cases enrolled in the NCT/DKTK MASTER trial with available RNA‐seq data. Patients' characteristics are reported in Table 3.

**Table 3:** Patient characteristics of the MASTER BTC cohort according to MGMT expression (TPM value). The *P* value of the χ^2^ test, Fisher's exact test (for categorical variables), or Mann–Whitney test (for continuous variables) assessing the association between each characteristic and MGMT status is indicated in the right column of the table. MASTER: Molecularly Aided Stratification for Tumor Eradication Research; eCCA: extrahepatic cholangiocarcinoma; iCCA: intrahepatic cholangiocarcinoma; IQR: interquartile range; MGMT: O6‐methylguanine DNA methyltransferase; NA: not available; NOS: not otherwise specified.

Characteristic	Total, *N* (%)	MGMT high, *N* (%)	MGMT low, *N* (%)	*P* value
Total number of patients	*N* = 76	*N* = 38	*N* = 38	
Age in years [median (IQR)]	47 (38–50)	45 (35–49)	48 (44–52)	0.079
Gender				
Female	31 (40.8)	17 (44.7)	14 (36.8)	0.641
Male	45 (59.2)	21 (55.3)	24 (63.2)	
Primary tumor location				
CCA NOS	7 (9.2)	4 (10.5)	3 (7.9)	0.937
iCCA	44 (57.9)	22 (57.9)	22 (57.9)	
eCCA	16 (21.1)	7 (18.4)	9 (23.7)	
Gallbladder	9 (11.8)	5 (13.2)	4 (10.5)	
Primary tumor resected				
No	40 (52.6)	18 (47.4)	22 (57.9)	0.491
Yes	36 (47.4)	20 (52.6)	16 (42.1)	
Adjuvant treatment				
Yes	9 (11.8)	3 (7.9)	6 (15.8)	0.230
No	27 (35.5)	17 (44.7)	10 (26.3)	
Not applicable	40 (52.6)	18 (47.4)	22 (57.9)	
Total lines of treatment for unresectable/metastatic disease				
1	19 (26.4)	11 (30.6)	8 (22.2)	0.687
2	19 (26.4)	8 (22.2)	11 (30.6)	
> 2	34 (47.2)	17 (47.2)	17 (47.2)	
NA	4	2	2	
First line treatment regimen				
Capecitabine/5‐FU	1 (1.9)	1 (3.8)	/	0.554
Capecitabine/5‐FU + Oxaliplatin	4 (7.5)	2 (7.7)	2 (7.4)	
Gemcitabine	2 (3.8)	1 (3.8)	1 (3.7)	
Gemcitabine + Cisplatin	35 (66.0)	19 (73.1)	16 (59.3)	
Gemcitabine + Oxaliplatin	2 (3.8)	1 (3.8)	1 (3.7)	
Other	9 (17.0)	2 (7.7)	7 (25.9)	
NA	23	12	11	
Platinum‐based first line regimen				
Yes	49 (92.4)	24 (92.3)	25 (92.6)	1.000
No	4 (7.5)	2 (7.7)	2 (7.4)	
NA	23	12	11	
MGMT promoter methylation status				
Yes	5 (10.6)	3 (12.5)	2 (8.7)	1.000
No	42 (89.4)	21 (87.5)	21 (91.3)	
NA	29	14	15	

Cases were grouped according to the median MGMT mRNA expression value (27.9). No significant differences were observed between patients with high and low MGMT expression in terms of baseline characteristics. Methylation data were available for 47 (62%) cases, of which 5 (11%) had *MGMT* promoter hypermethylation. No association between MGMT expression and promoter methylation was observed (Wilcoxon *P* = 0.44, Fig. S9).

At data cut off, the median follow‐up was 39.4 months (18.0–66.5) and the median OS was 25.4 months (IQR 16.4–60.7); 53 (70%) patients received first‐line chemotherapy, with a median 1L‐PFS of 5.0 months (IQR 3.0–9.0); median 1L‐PFS and mOS were not affected by use of platinum in 1‐line chemotherapy (mPFS 5.0 vs 5.7, *P* = 0.8; mOS 20.9 vs 71.4, *P* = 0.1).

**Fig. 3:**
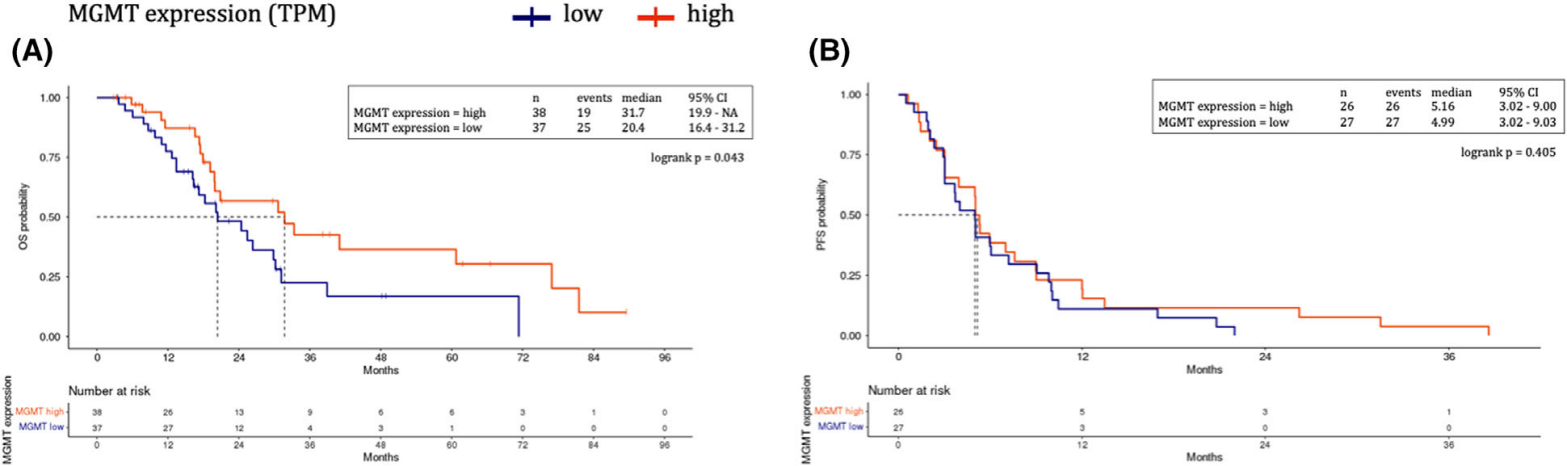
MGMT expression assessed by RNAseq (TPM values). Overall survival (OS) (A) and first‐line progression‐free survival (B) represented through Kaplan–Meier curves according to MGMT expression in the MASTER BTC cohort. Patients with lower MGMT mRNA expression (blue) showed significantly poorer OS as compared with patients with high MGMT expression (red) (A). MGMT mRNA expression had no significant impact on progression‐free survival of of patients treated with first‐line systemic treatment (B). Dotted lines indicate the median survival time. MGMT, *O* ^6^‐methylguanine‐DNA methyltransferase; TPM, transcripts per kilobase million; RNAseq, RNA sequence; MASTER BTC Molecularly Aided Stratification For Tumor Eradication Research on Biliary Tract Cancers. [Colour figure can be viewed at wileyonlinelibrary.com]

Patients with lower MGMT mRNA expression showed significantly poorer OS (mOS 20.4 vs 31.7 months, unadjusted HR 1.89; 95% CI 1.01–3.56; *P* = 0.043), which was confirmed in a multivariable model accounting for patients' age, gender, and primary tumor location, prior tumor resection and use of adjuvant therapy (Fig. 3A and Table S4). For 1L‐PFS, MGMT mRNA expression showed no significant impact (mPFS 5.0 vs 5.2 months, unadjusted HR 1.27; 95% CI 0.72–2.22; *P* = 0.405), and a multivariate model showed only a trend for interaction (*P* = 0.115) between MGMT expression and use of platinum‐based chemotherapy (Fig. 3B and Table S5). Concerning DNA methylation data, no reliable association with survival outcomes could be observed, both in terms of OS and PFS, as this analysis was limited by the low number of cases and events (*n* = 2) in the subgroup of patients with MGMT promoter hypermethylation.

### 3.3 Case series of temozolomide‐treated BTC patients

Based on the published clinical activity of TMZ in *MGMT* methylated colorectal cancers [18, 20], four patients with no other therapeutic options were treated at INT with TMZ‐based therapies from December 2018 until September 2019. Among these, two patients were treated with TMZ plus irinotecan (TEMIRI regimen) [18], while two patients received single‐agent TMZ. The clinical characteristics of these patients are detailed in Table S6. Of note, all but one cases were affected by iCCA, and all were previously treated with at least one platinum‐based chemotherapy. Median *MGMT* promoter methylation value was 12 (range 9–49), and three patients were evaluated for MGMT expression by IHC, all resulting negative. Figure S10 shows the swimmer plot of PFS during TMZ‐based treatment: three patients had stable disease as best response, with a PFS ranging from 2.0 to 6.1 months overall. No new safety signals were observed.

## 4 Discussion

Here we showed that both a high *MGMT* promoter hypermethylation and low/absent MGMT expression are associated with worse OS in patients with advanced BTC. Our evidence was consistently validated in two independent cohorts of patients and by investigating different assays including methylation‐specific (MSP) PCR, IHC, and RNA‐seq. Previous studies investigated the frequency of *MGMT* promoter hypermethylation in BTC [29, 30] and its role in tumor progression [31, 32] with inconclusive results, mostly due to the small sample size, the lack of clinical information, and the variability of assays used for testing this biomarker. Whether MGMT inactivation may be associated with a more aggressive behavior and which are the mechanisms causing this is not yet fully elucidated. However, MGMT loss is linked with an increased susceptibility to acquiring other mutations in both oncogenes and tumor suppressor genes, thus potentially stimulating tumor progression and poorer prognosis [17].

The prevalence of *MGMT* promoter hypermethylation in the Italian cohort was 18%, which is clinically relevant in these rare cancers. Furthermore, despite the over‐representation of iCCAs in the Italian cohort, we found a greater proportion of *MGMT* hyper‐methylated samples in eCCA and GBC. Given the heterogeneity of the disease, there is an unmet need for new therapeutic targets, especially for patients lacking targeted options such as those with eCCA and GBC, since most of the known actionable molecular alterations are usually found in iCCA. Therefore, our finding is potentially important from a clinical point of view, given the increasing body of evidence on efficacy for TMZ‐based regimens in *MGMT*‐methylated gastrointestinal tumors and especially colorectal cancer [19, 20, 33, 34]. TMZ may indeed be considered as a ‘targeted chemotherapy’ and even an agnostic investigational option in cancers with MGMT inactivation.

*MGMT* hypermethylation did not show an impact in terms of 1L‐PFS, probably due to the heterogeneity of treatments administered in this retrospective series. However, we found a significant interaction between the use of platinum‐based chemotherapy and *MGMT* promoter hypermethylation, despite these data should be interpreted with caution, given the non‐randomized nature of our treatment groups. From a biological perspective, our data are consistent with the growing evidence suggesting that *MGMT* may be involved in platinum‐induced DNA damage response (DDR) by playing a role in the homologous recombination signaling in cancer cells [35]. The latter consideration is particularly interesting given the amount of new evidence regarding possible combinations of TMZ with DDR inhibitors such as Poly‐ADP‐ribose polymerase (PARP) [36, 37] and ataxia telangiectasia mutated and Rad3‐related (ATR) inhibitors [38]. Furthermore, various preclinical and clinical observations have linked acquired resistance to TMZ to the emergence of alterations in the mismatch repair system and increased tumor mutational burden, that could be exploited in combinations of TMZ and immune checkpoint inhibitors [21, 39]. Here, we reported four cases that were treated with TMZ‐based regimens, with three patients experiencing stable disease as best response. Though we cannot derive conclusions from such a small number of patients, these patients were heavily pretreated and not strictly selected according to a *MGMT* methylation cutoff, thus highlighting the opportunity to better investigate the potential activity of TMZ in molecularly hyper‐selected subgroups and as part of combination regimens with potentially synergic drugs [18, 40]. Interestingly, the patient achieving the longest PFS had a lower methylation value compared to others; in this regard, the results may be explained by the fact that tumor MGMT expression was negative, other than that the patient was less pretreated than the others and received a combination regimen (TEMIRI).

In the Italian cohort, we explored whether any molecular alteration was enriched in cases with MGMT inactivation, but no clear pattern was observed. The lack of significant association with *IDH1/2* mutations in the high *MGMT* methylation group is somewhat unexpected, given that these mutations ultimately lead to the formation of oncometabolite 2‐hydroxyglutarate (2HG), which has been associated with hypermethylator phenotype [CpG island methylator phenotype (CIMP)] and MGMT silencing [41, 42, 43, 44, 45]. However, given the overall rarity of individual molecular alterations and the non‐uniformity of the molecular characterization performed in this cohort, further studies on larger datasets are required.

Our analysis has limitations, mainly originating in the retrospective nature of the investigation in both cohorts, which included heterogeneous series of patients in terms of primary tumor origin, molecular profiling, and adopted treatments. Concerning the Italian cohort, limited evidence could be derived from IHC analysis, which was performed only on ~ 60% of cases. As for the MASTER BTC cohort, OS was available from the first tumor diagnosis instead of diagnosis of advanced disease, data regarding the classification of different subtypes of eCCA (pCCA and dCCA) were not available and only a small number of cases were profiled with methylation analysis, thus limiting the evidence derived from this data layer.

Both cohorts included only patients with advanced disease, either at initial diagnosis or at metachronous relapse after surgery. However, while the Italian cohort also included patients with rapidly progressing disease and treated with best supportive care, as per clinical practice, all patients in the MASTER BTC cohort were pretreated with standard chemotherapy options and had optimal ECOG PS at enrolment. Of note, highly methylated cases in the Italian cohort more frequently had worse baseline ECOG PS and did not receive first‐line platinum‐based chemotherapy. This does not seem to influence the results of our study, since *MGMT* status was confirmed as an independent negative prognostic factor for OS in the multivariable models that considered other prognostic variables in both cohorts. Overall, despite these differences, the two cohorts are well representative of this rare tumor entity: median 1L‐PFS was in line with literature and real‐life data, while the OS is coherent with the availability of new treatment options in later lines of treatment and the patients' selection that inevitably occurs in high‐volume cancer centers. While detailed data about later lines of treatment are not available, 26% and 47% of Italian and German patients were treated with more than two lines, respectively, and this likely had an impact on the OS.

Finally, in both cohorts, *MGMT* promoter methylation and expression did not show an optimal concordance, as previously reported in other works [17]. There is no unique explanation to the discordance between methylation and protein expression results and the regulation of *MGMT* inactivation is far from being fully clarified. Methylation is probably not the only mechanisms behind MGMT protein silencing, as several transcription factors, such as secreted protein 1 (SP1), CCAAT‐enhancer‐binding proteins (CEBP), activator protein 1 (AP1), hypoxia inducible factor‐1α (HIF‐1α), and the p65 (RELA) subunit of the nuclear factor kappa‐light‐chain‐enhancer of activated B cells (NF‐κB) [46], bind to the MGMT promoter to induce or suppress MGMT expression [47]. Furthermore, *MGMT* promoter CpG islands may present a differential pattern of methylation along the region, with some CpGs being more important in terms of gene transcription and correlation with protein expression. Finally, methylation data in both cohorts may be influenced by contamination of the tumor sample from surrounding normal tissue, and spatial and temporal tumoral heterogeneity may influence the results of IHC.

Given these considerations, our findings are in line with previous evidence, suggesting that these methodologies should be used as complementary and not interchangeably in clinical practice [17] and that IHC could be regarded as a method for refining the discriminative ability of MGMT assessment in terms of survival. This approach has shown promising results in metastatatic colorectal cancer, as proved by the recently published MAYA trial [21].

Overall, despite these limitations, in these large series of BTCs cases treated at three European comprehensive cancer centers and tested with three different methodologies, our results are a convincing proof of concept of the negative prognostic impact of MGMT inactivation in BTCs.

## 5 Conclusions

We provide the first clear evidence that *MGMT* inactivation is a frequent epigenetic alteration in BTC patients, with a relevant prognostic impact. Based on our data, we believe that *MGMT* is indeed a new piece of the molecular puzzle of BTCs and could serve to prospectively explore the efficacy of alkylating agents in *MGMT*‐silenced BTCs in future trials, possibly in combinations with DNA damaging agents or novel DDR inhibitors.

## Conflict of interest

MN: Travel expenses from Celgene, speaker honorarium from Accademia della Medicina; honoraria from Sandoz, Medpoint SRL for editorial collaboration. Consultant honoraria from EMD Serono, Basilea Pharmaceutica, Incyte and MSD Italia. FM: honoraria from SERVIER, research grant from Incyte. SP: Consultant/Advisory role: Ipsen, Novartis, Pfizer, Advanced accelerator Application AAA, Merk. GP: honoraria from Foundation Medicine. FP: honoraria from Amgen, Roche, Sanofi, Bayer, Servier, Merck‐Serono, Lilly, MSD, Astrazeneca; advisory role with Amgen, Bayer, Servier, Merk‐Serono, MSD and research grants from Bristol‐Myers Squibb, AstraZeneca and Incyte. AIRC under IG 2019 – ID. 23 624 project – P.I. Pietrantonio Filippo. MDB: honoraria from Lilly, MSD Oncology, Servier, consulting/advisory role with Lilly, MSD oncology, research grant from Lilly, travel/accommodation expenses from Roche and Sanofi. SF: Consulting or advisory board membership: Bayer, Illumina, Roche; honoraria: Amgen, Eli Lilly, PharmaMar, Roche; research funding: AstraZeneca, Pfizer, PharmaMar, Roche; travel or accommodation expenses: Amgen, Eli Lilly, Illumina, PharmaMar, Roche. FdB: Consultant Advisory Board: Roche, EMD Serono, NMS Nerviano Medical Science, Sanofi, MSD, Novartis, Incyte, BMS, Menarini. Speaker: BMS, Healthcare Research & Pharmacoepidemiology, Merck Group, ACCMED, Nadirex, MSD, Pfizer, Servier, Sanofi, Roche, AMGEN, Incyte, Dephaforum. Principal Investigator for Novartis, F.Hoffmann‐LaRoche Ltd, BMS, Ignyta Operating INC, Merck Sharp & Dohme Spa, Kymab, Pfizer, Tesaro, MSD, MedImmune LCC, Exelixis Inc., LOXO Oncology Incorporated, DAICHI SANKIO Dev. Limited, Basilea Pharmaceutica International AG, Janssen‐Cilag International NV, Merck KGAA. All other authors declare no conflict of interest.

## Author contributions

MN, FdB, MDB, FN, FP: Study concept and design; All authors: Acquisition of data; MN, FN, JH, AD, DBL, FdB, FP: Analysis and interpretation of data; MN, FN, DBL, FP, FM: Drafting of the manuscript; All authors: Manuscript revision and input; FN, LA, AV: Statistical analysis; MN, BCK, DBL, DH, SF, FdB: Study supervision.

## Supporting information


**Fig. S1.** Study flowchart for the Italian cohort.
**Fig. S2.** Graphical representation of Cox regression model evaluating MGMT promoter methylation impact on patients' OS, with non‐linear effects handled by restricted cubic splines (Italian cohort). MGMT, *O* ^6^‐methylguanine‐DNA methyltransferase; OS, overall survival.
**Fig. S3.** Graphical comparison of Minimal Depth and VIMP rankings for OS prediction. Covariates ranking in the lower left quadrant were included in multivariable Cox Proportional Hazard Model (Italian cohort). MGMT, *O* ^6^‐methylguanine‐DNA methyltransferase; VIMP, variable importance; OS, overall survival.
**Fig. S4.** Graphical representation of Cox regression model evaluating MGMT promoter methylation on impact on patients' 1L‐PFS, with non‐linear effects handled by restricted cubic splines (Italian cohort). 1L‐PFS; first‐line progression‐free survival; MGMT, *O* ^6^‐methylguanine‐DNA methyltransferase.
**Fig. S5**. Graphical comparison of Minimal Depth and VIMP rankings for PFS prediction. Covariates ranking in the lower left quadrant were included in multivariable Cox Proportional Hazard Model (Italian cohort). VIMP, variable importance; PFS, progression‐free survival.
**Fig. S6.** (a,b). Progression‐free survival represented through Kaplan–Meier curves according to use of platinum‐based chemotherapy (CT) in patients with high (>14%, 6a) and low (≤14%, 6b) *MGMT* promoter methylation status (Italian cohort). MGMT, *O* ^6^‐methylguanine‐DNA methyltransferase.
**Fig. S7.** (a,b). Overall survival and progression‐free survival represented through Kaplan–Meier curves according to MGMT status assessed by IHC reported on three levels. MGMT, *O* ^6^‐methylguanine‐DNA methyltransferase; IHC, immunohistochemistry.
**Fig. S8**. Oncoplot of molecular alterations in patients profiled with the IonTorrent® or FoundationOne®CDx panel in the INT cohort.
**Fig. S9**. MGMT mRNA expression (TPM values) according to MGMT promoter methylation in the MASTER BTC cohort. MASTER BTC Molecularly Aided Stratification For Tumor Eradication Research on Biliary Tract Cancers; MGMT, *O* ^6^‐methylguanine‐DNA methyltransferase; TPM, transcripts per kilobase million.
**Fig. S10**. Swimmer plot of patients treated with temozolomide‐based regimens. MGMT promoter methylation values are reported right to each patients' bar. MGMT, *O* ^6^‐methylguanine‐DNA methyltransferase.
**Table S1.** Patients' characteristics according to MGMT promoter methylation (with the 14% cutoff) in the Italian cohort. MGMT, *O* ^6^‐methylguanine‐DNA methyltransferase.
**Table S2.** Multivariable Cox proportional hazards model for progression‐free survival in the Italian cohort. Missing data of covariates included in the model were imputed.
**Table S3.** Patients' characteristics according to MGMT expression by IHC in the Italian cohort. MGMT, *O* ^6^‐methylguanine‐DNA methyltransferase; IHC, immunohistochemistry.
**Table S4.** Multivariable Cox proportional hazards model for overall survival in the MASTER BTC cohort. MASTER BTC Molecularly Aided Stratification For Tumor Eradication Research on Biliary Tract Cancers.
**Table S5.** Multivariable Cox proportional hazards model for progression‐free survival in the MASTER BTC cohort. MASTER BTC Molecularly Aided Stratification For Tumor Eradication Research on Biliary Tract Cancers.
**Table S6.** Clinical characteristics of patients treated with temozolomide.

## Data Availability

Clinical data of the Italian and German cohorts were collected through an electronic database and are available upon reasonable request to the corresponding author. Sequencing data of the MASTER program have been deposited in the European Genome‐phenome Archive (https://www.ebi.ac.uk/ega/datasets) under accession EGAS00001004813.
