# Supplementary material for: MGMT inactivation as a new biomarker in patients with advanced biliary tract cancers
Source: Mol Oncol. 2022 Jun 13;16(14):2733–46. doi: 10.1002/1878-0261.13256 (PMC9297767; doi:10.1002/1878-0261.13256)
Supplement: Supplementary file 1 — Fig. S1. Study flowchart for the Italian cohort. Fig. S2. Graphical representation of Cox regression model evaluating MGMT promoter methylation impact on patients' OS, with non‐linear effects handled by restricted cubic splines (Italian cohort). MGMT, O 6‐methylguanine‐DNA methyltransferase; OS, overall survival. Fig. S3. Graphical comparison of Minimal Depth and VIMP rankings for OS prediction. Covariates ranking in the lower left quadrant were included in multivariable Cox Proportional Hazard Model (Italian cohort). MGMT, O 6‐methylguanine‐DNA methyltransferase; VIMP, variable importance; OS, overall survival. Fig. S4. Graphical representation of Cox regression model evaluating MGMT promoter methylation on impact on patients' 1L‐PFS, with non‐linear effects handled by restricted cubic splines (Italian cohort). 1L‐PFS; first‐line progression‐free survival; MGMT, O 6‐methylguanine‐DNA methyltransferase. Fig. S5. Graphical comparison of Minimal Depth and VIMP rankings for PFS prediction. Covariates ranking in the lower left quadrant were included in multivariable Cox Proportional Hazard Model (Italian cohort). VIMP, variable importance; PFS, progression‐free survival. Fig. S6. (a,b). Progression‐free survival represented through Kaplan–Meier curves according to use of platinum‐based chemotherapy (CT) in patients with high (>14%, 6a) and low (≤14%, 6b) MGMT promoter methylation status (Italian cohort). MGMT, O 6‐methylguanine‐DNA methyltransferase. Fig. S7. (a,b). Overall survival and progression‐free survival represented through Kaplan–Meier curves according to MGMT status assessed by IHC reported on three levels. MGMT, O 6‐methylguanine‐DNA methyltransferase; IHC, immunohistochemistry. Fig. S8. Oncoplot of molecular alterations in patients profiled with the IonTorrent® or FoundationOne®CDx panel in the INT cohort. Fig. S9. MGMT mRNA expression (TPM values) according to MGMT promoter methylation in the MASTER BTC cohort. MASTER BTC Molecularly Aided Stratification Fo [file MOL2-16-2733-s001.docx]

**Supplementary Tables**

**Supplementary Table 1**. Patients’ characteristics according to MGMT promoter methylation (with the 14% cutoff) in the Italian cohort.

| Characteristic | MGMT ≤ 14%  N (%) | MGMT > 14%  N (%) | p |
| --- | --- | --- | --- |
| Total number of patients | **N = 135** | **N = 29** |  |
| Age in years [median (IQR)] | 64 (57 - 71) | 71 (64 - 81) | 0.003 |
| Gender  Male  Female | 59 (43.7)  76 (56.3) | 12 (41.4)  17 (58.6) | 0.819 |
| ECOG PS  0 - 1  ≥ 2  NA | 111 (91.0)  11 (9.0)  13 | 17 (60.7)  11 (39.3)  1 | < .001 |
| Primary tumor location  iCCA  pCCA  dCCA  Gallbladder | 90 (66.7)  7 (5.2)  17 (12.6)  21 (15.6) | 10 (34.5)  -  6 (20.7)  13 (44.8) | < .001 |
| Primary tumor resected  Yes  No | 84 (62.2)  51 (37.8) | 23 (79.3)  6 (20.7) | 0.080 |
| Adjuvant treatment  Yes  No  Not applicable | 36 (26.7)  48 (35.6)  51 (37.8) | 10 (34.5)  13 (44.8)  6 (20.7) | 0.215 |
| Diagnosis of advanced disease*  Synchronous  Metachronous | 67 (49.6)  68 (50.4) | 18 (62.1)  11 (37.9) | 0.224 |
| Liver-limited disease  Sites of metastatic disease  Lymph nodes  Bones  Liver  Lungs  Peritoneum | 39 (28.9)  69 (51.1)  9 (6.7)  74 (54.8)  24 (17.8)  26 (19.3) | 9 (31.0)  9 (31.0)  2 (6.9)  16 (55.2)  10 (34.5)  5 (17.2) | 0.818  0.050  1.000  0.972  0.044  1.000 |
| Total lines of treatment for unresectable/metastatic disease  Best Supportive Care  1  2  >2  NA | 3 (2.5)  41 (34.2)  39 (32.5)  37 (30.8)  15 | 1 (11.1)  2 (22.2)  1 (11.1)  5 (55.6)  20 | 0.131 |
| First line treatment regimen  Best Supportive Care  Capecitabine/5-FU  Gemcitabine     Capecitabine/5-FU + Oxaliplatin     Gemcitabine + Oxaliplatin     Gemcitabine + Cisplatin     Other  NA | 3 (2.3)  5 (3.8)  11 (8.4)  11 (8.4)  11 (8.4)  80 (61.1)  10 (7.6)  4 | 1 (3.4)  7 (24.1)  1 (3.4)  4 (13.8)  -  8 (27.6)  8 (27.6)  - | < .001 |
| Platinum-based first line regimen  Yes  No  NA | 103 (80.5)  25 (19.5)  7 | 12 (42.9)  16 (57.1)  1 | < .001 |
| MGMT expression by IHC  Positive  Weakly positive  Negative  NA | 25 (26.9)  32 (34.4)  36 (38.7)  42 | 2 (28.6)  /  5 (71.4)  22 | 0.105 |
| * Diagnosis of advanced disease was considered synchronous if occurring <6 months from primary tumor detection, metachronous if ≥6 months.  The p value of the χ2 test, Fisher’s exact test (for categorical variables) or Mann-Whitney test (for continuous variables) assessing the association between each characteristic and MGMT status is indicated in the right column of the table.  Abbreviations: 5-FU: 5-fluorouracil; dCCA: distal cholangiocarcinoma; ECOG PS: Eastern Cooperative Oncology Group Performance Status; iCCA: intrahepatic cholangiocarcinoma; IHC: immunohistochemistry; IQR: interquartile range; MGMT: O6-methylguanine DNA methyltransferase; NA: not available; pCCA: perihilar cholangiocarcinoma. | | | |

**Supplementary Table 2.** Multivariable Cox proportional hazards model for Progression Free Survival in the Italian cohort. Missing data of covariates included in the model were imputed.

| Variables |  | HR | 95% CI | p |
| --- | --- | --- | --- | --- |
| *MGMT* promoter methylation | High (>14%) vs low (≤14%) | 2.43 | 1.08 - 5.48 | 0.038* |
| Platinum-based first line regimen | Yes vs no | 0.92 | 0.57 - 1.48 | 0.038* |
| Age | Continuous | 1.02 | 0.99 - 1.04 | 0.055 |
| Adjuvant treatment | Not applicable (no tumor resection) vs no  Yes vs no | 1.29  1.07 | 0.85 - 1.95  0.67 - 1.71 | 0.233  0.767 |
| Presence of lung metastases | Yes vs No | 1.46 | 0.93 - 2.29 | 0.101 |
| The HR for continuous variables is expressed as the HR variation per unit increase of the variable value (i.e. per 1 year increase).  * p for interaction  *Abbreviations*: CI: confidence interval; HR: Hazard Ratio; MGMT: O6-methylguanine DNA methyltransferase. | | | | |

**Supplementary Table 3**. Patients’ characteristics according to MGMT expression by IHC in the Italian cohort.

| Characteristic | Total  N (%) | MGMT IHC positive  N (%) | MGMT IHC negative  N (%) | p |
| --- | --- | --- | --- | --- |
| Total number of patients | N = 100 | N = 27 | N = 73 |  |
| Age in years [median (IQR)] | 62 (56 - 70) | 57 (52 - 62) | 65 (58 - 71) | <.001 |
| Gender  Male  Female | 49 (49.0)  51 (51.0) | 13 (48.1)  14 (51.9) | 36 (49.3)  37 (50.7) | 0.917 |
| ECOG PS  0 - 1  ≥ 2  NA | 81 (92.0)  7 (8.0)  12 | 19 (90.5)  2 (9.5)  6 | 62 (92.5)  5 (7.5)  6 | 0.670 |
| Primary tumor location  iCCA  pCCA  dCCA  Gallbladder | 71 (71.0)  5 (5.0)  14 (14.0)  10 (10.0) | 20 (74.1)  3 (11.1)  2 (7.4)  2 (7.4) | 51 (69.9)  2 (2.7)  12 (16.4)  8 (11.0) | 0.257 |
| Primary tumor resected  Yes  No | 60 (60.0)  40 (40.0) | 14 (51.9)  13 (48.1) | 46 (63.0)  27 (37.0) | 0.312 |
| Adjuvant treatment  Yes  No  Not applicable | 24 (24.0)  36 (36.0)  40 (40.0) | 7 (25.9)  7 (25.9)  13 (48.1) | 17 (23.3)  29 (39.7)  27 (37.0) | 0.424 |
| Diagnosis of advanced disease*  Synchronous  Metachronous | 50 (50.0)  50 (50.0) | 16 (59.3)  11 (40.7) | 34 (46.6)  39 (53.4) | 0.260 |
| Liver-limited disease  Sites of metastatic disease  Lymph nodes  Bones  Liver  Lungs  Peritoneum | 25 (34.2)  55 (55.0)  6 (6.0)  55 (55.0)  13 (13.0)  21 (21.0) | 29 (29.0)  18 (66.7)  3 (11.1)  15 (55.6)  3 (11.1)  4 (14.8) | 4 (14.8)  37 (50.7)  3 (4.1)  40 (54.8)  10 (13.7)  17 (23.3) | 0.082  0.154  0.3390.946  0.999  0.420 |
| Total lines of treatment for unresectable/metastatic disease  Best Supportive Care  1  2  >2  NA | 1 (1.0)  33 (34.4)  30 (31.2)  32 (33.3)  4 | -  10 (37.0)  6 (22.2)  11 (40.7)  - | 1 (1.4)  23 (33.3)  24 (34.8)  21 (30.4)  4 | 0.623 |
| First line treatment regimen  Best Supportive Care  Capecitabine/5-FU  Gemcitabine     Capecitabine/5-FU + Oxaliplatin     Gemcitabine + Oxaliplatin     Gemcitabine + Cisplatin     Other  NA | 1 (1.0)  5 (5.2)  8 (8.3)  7 (7.3)  9 (9.4)  63 (65.6)  3 (3.1)  4 | -  1 (3.7)  2 (7.4)  1 (3.7)  3 (11.1)  18 (66.7)  2 (7.4)  - | 1 (1.4)  4 (5.8)  6 (8.7)  6 (8.7)  6 (8.7)  45 (65.2)  1 (1.4)  4 | 0.791 |
| Platinum-based first line regimen  Yes  No  NA | 79 (83.2)  16 (16.8)  5 | 22 (81.5)  5 (18.5)  - | 57 (83.8)  11 (16.2)  5 | 0.768 |
| *MGMT* promoter methylation  [median (IQR)] | 3.5 (2.0 - 7-0) | 4.0 (1.5 - 5.5) | 3.0 (3.0 - 7.0) | 0.594 |
| * Diagnosis of advanced disease was considered synchronous if occurring <6 months from primary tumor detection, metachronous if ≥6 months.  The p value of the χ2 test, Fisher’s exact test (for categorical variables) or Mann-Whitney test (for continuous variables) assessing the association between each characteristic and MGMT-IHC status is indicated in the right column of the table.  Abbreviations: 5-FU: 5-fluorouracil; dCCA: distal cholangiocarcinoma; ECOG PS: Eastern Cooperative Oncology Group Performance Status;; iCCA: intrahepatic cholangiocarcinoma; IHC: immunhistochemistry; IQR: interquartile range; MGMT: O6-methylguanine DNA methyltransferase; NA: not available; pCCA: perihilar cholangiocarcinoma. | | | | |

**Supplementary Table 4.** Multivariable Cox proportional hazards model for Overall Survival in the MASTER BTC cohort.

| Variables |  | HR | 95% CI | p |
| --- | --- | --- | --- | --- |
| MGMT expression (TPM) | Low (below median) vs  high (above median) | 2.07 | 1.06 - 4.04 | 0.034 |
| Gender | Male vs Female | 0.86 | 0.45 - 1.63 | 0.644 |
| Age | Continuous | 0.97 | 0.93 - 1.01 | 0.117 |
| Primary Tumor Location | iCCA vs CCA NOS  eCCA vs CCA NOS  Gallbladder vs CCA NOS | 0.37  0.54  0.57 | 0.13 - 1.09  0.16 - 1.78  0.16 - 2.01 | 0.070  0.310  0.383 |
| Primary tumor resected | Yes vs no | 0.15 | 0.06 - 0.35 | <.001 |
| Adjuvant treatment | Yes vs no | 1.27 | 0.36 - 4.51 | 0.710 |
| The HR for continuous variables is expressed as the HR variation per unit increase of the variable value (i.e. per 1 year increase).  *Abbreviations*: CI: confidence interval; eCCA: extrahepatic cholangiocarcinoma; iCCA: intrahepatic cholangiocarcinoma; HR: Hazard Ratio; MGMT: O6-methylguanine DNA methyltransferase; NOS: not otherwise specified; TPM: transcripts per million. | | | | |

**Supplementary Table 5.** Multivariable Cox proportional hazards model for Progression Free Survival in the MASTER BTC cohort.

| Variables |  | HR | 95% CI | p |
| --- | --- | --- | --- | --- |
| MGMT expression (TPM) | Low (below median) vs  high (above median) | 0.30 | 0.04 - 2.33 | 0.115* |
| Platinum-based first line regimen | Yes vs no | 0.20 | 0.04 - 1.01 | 0.115* |
| Gender | Male vs Female | 1.09 | 0.58 – 2.04 | 0.795 |
| Age | Continuous | 0.94 | 0.90 - 0.98 | 0.003 |
| Primary Tumor Location | iCCA vs CCA NOS  eCCA vs CCA NOS  Gallbladder vs CCA NOS | 0.17  0.50  0.43 | 0.05 - 0.66  0.12 - 1.89  0.10 - 1.87 | 0.011  0.308  0.259 |
| Primary tumor resected | Yes vs no | 0.45 | 0.22 - 0.89 | 0.023 |
| Adjuvant treatment | Yes vs no | 6.15 | 1.49 - 25.5 | 0.012 |
| The HR for continuous variables is expressed as the HR variation per unit increase of the variable value (i.e. per 1 year increase).  *p for interaction  *Abbreviations*: CI: confidence interval; eCCA: extrahepatic cholangiocarcinoma; iCCA: intrahepatic cholangiocarcinoma; HR: Hazard Ratio; MGMT: O6-methylguanine DNA methyltransferase; NOS: not otherwise specified; TPM: transcripts per million. | | | | |

**Supplementary Table 6**. Clinical characteristics of patients treated with temozolomide.

| Characteristic | Patient 1 | Patient 2 | Patient 3 | Patient 4 |
| --- | --- | --- | --- | --- |
| Age, years | 54 | 73 | 81 | 68 |
| Gender | Female | Male | Male | Male |
| ECOG PS | 0 | 1 | 1 | 1 |
| Primary tumor location | iCCA | iCCA | iCCA | Gallbladder |
| Primary tumor resected | No | Yes | No | yes |
| Adjuvant treatment | Not applicable | No | Not applicable | No |
| Metastases | Synchronous | Metachronous | Synchronous | Metachronous |
| Sites of metastatic disease | Liver | Liver,  Lymph nodes,  Lung | Liver,  Lymph nodes | Liver,  Lung,  Peritoneum |
| Lines of treatment for unresectable/metastatic disease | >2 | >2 | >2 | 2 |
| First line treatment regimen | Gemcitabine + Cisplatin | Gemcitabine + Cisplatin | 5-FU + Oxaliplatin | Gemcitabine + Oxaliplatin |
| MGMT promoter methylation | 9 | 12 | 49 | 12 |
| MGMT expression by IHC | negative | negative | - | negative |
| Treatment Regimen | TEMIRI | temozolomide | temozolomide | TEMIRI |
| Line of temozolomide-based treatment | 2 | 5 | 3 | 3 |
| Best response on temozolomide | SD | SD | SD | PD |
| Progression Free Survival on temozolomide (months) | 6.1 | 3.7 | 3.0 | 2.0 |
| Grade 1 - 2 toxicities | Nausea G1 | Diarrhea G2, Fatigue G2, Anemia G2 | - | Nausea G1, Constipation G1, Platelet count decreased G1, Abdominal Pain G1, Paresthesia G1, Fever G2, Fatigue G2, Anemia G2 |
| Grade 3 toxicities | - | - | Fatigue | - |
| *Abbreviations*: 5-FU: 5-fluorouracil; ECOG PS: Eastern Cooperative Oncology Group Performance Status; G: grade; iCCA: intrahepatic cholangiocarcinoma; IHC: immunohistochemistry; MGMT: O6-methylguanine DNA methyltransferase; NA: not available; PD: progressive disease; SD: stable disease; TEMIRI: temozolomide plus irinotecan. | | | | |

**Supplementary Figures**

**Supplementary Figure 1.** Study flowchart for the Italian cohort.

*Abbreviations*: IHC: immunohistochemistry; MGMT: O6-methylguanine DNA methyltransferase; FFPE: formalin-fixed paraffin-embedded.

**Supplementary Figure 2.** Graphical representation of Cox regression model evaluating *MGMT* promoter methylation impact on patients’ OS, with non-linear effects handled by restricted cubic splines (Italian cohort).

*Abbreviations*: MGMT: O6-methylguanine DNA methyltransferase; OS: Overall Survival.

**
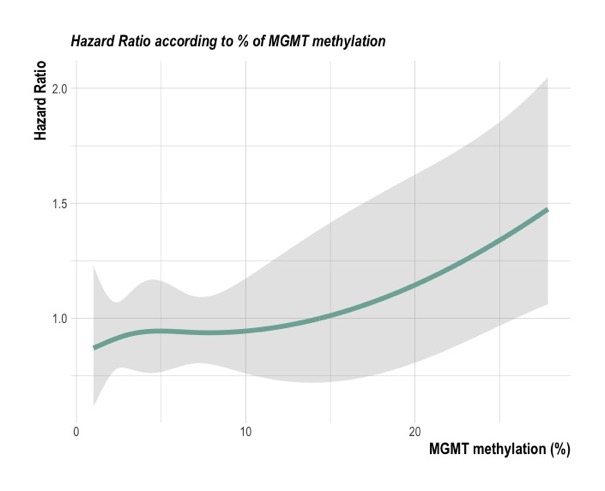
**

**Supplementary Figure 3**. Graphical comparison of Minimal Depth and VIMP rankings for OS prediction. Covariates ranking in the lower left quadrant were included in multivariable Cox Proportional Hazard Model (Italian cohort).

*Abbreviations*: ECOG PS: Eastern Cooperative Oncology Group Performance Status; MGMT: O6-methylguanine DNA methyltransferase; OS: Overall Survival; VIMP: Variable Importance statistic; Tx: treatment.


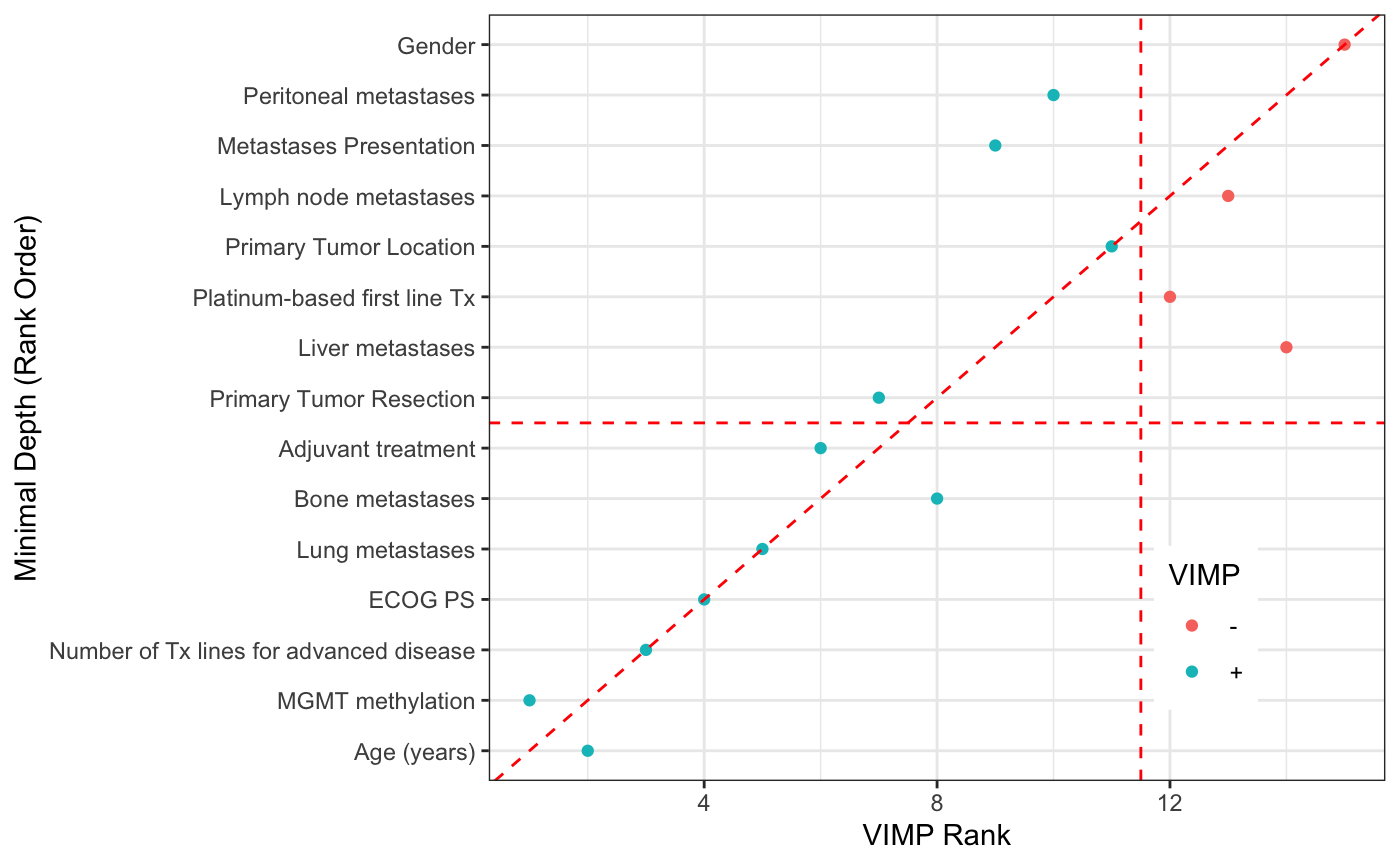


**Supplementary Figure 4.** Graphical representation of Cox regression model evaluating *MGMT* promoter methylation on impact on patients’ 1L-PFS, with non-linear effects handled by restricted cubic splines (Italian cohort).

*Abbreviations*: MGMT: O6-methylguanine DNA methyltransferase; 1L-PFS: First-Line Progression Free Survival.


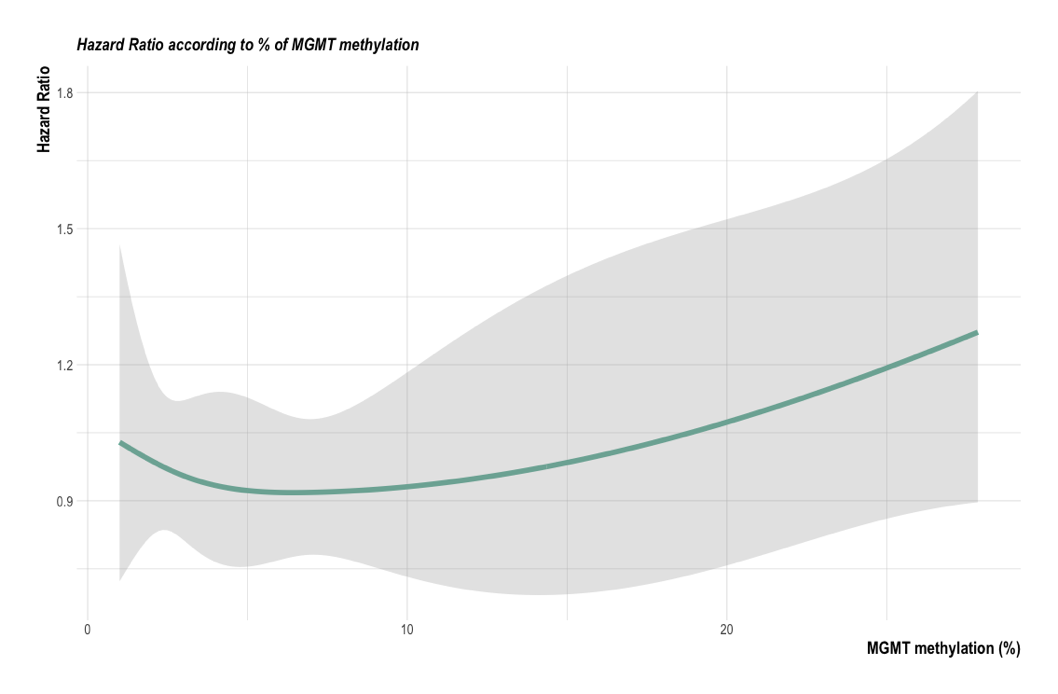


**Supplementary Figure 5**. Graphical comparison of Minimal Depth and VIMP rankings for PFS prediction. Covariates ranking in the lower left quadrant were included in multivariable Cox Proportional Hazard Model (Italian cohort).

*Abbreviations*: ECOG PS: Eastern Cooperative Oncology Group Performance Status; MGMT: O6-methylguanine DNA methyltransferase; OS: Overall Survival; VIMP: Variable Importance statistic; Tx: treatment.


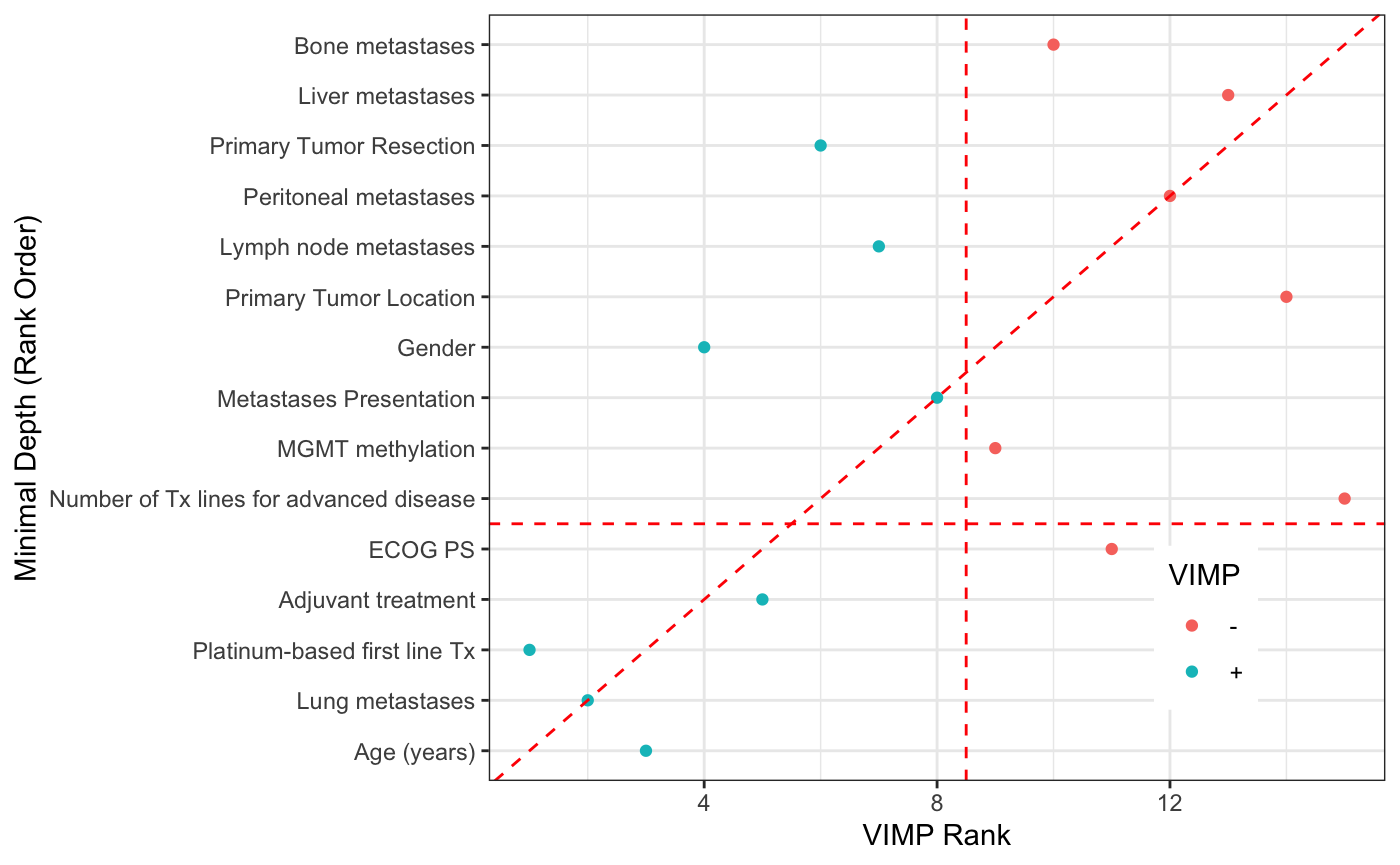


**Supplementary Figure 6 (a-b).** Progression Free Survival represented through Kaplan-Meier curves according to use of platinum-based CT in patients with high (>14%, 6a) and low (≤14%, 6b) MGMT promoter methylation status (Italian cohort).

*Abbreviations*: CT: chemotherapy; MGMT: O6-methylguanine DNA methyltransferase; PFS: Progression Free Survival.

**Supplementary Figure 7 (a-b).** Overall Survival and Progression Free Survival represented through Kaplan-Meier curves according to MGMT status assessed by IHC reported on three levels.

**** ****

**Supplementary Figure 8**. Oncoplot of molecular alterations in patients profiled with the IonTorrentⓇ or FoundationOne®CDx panel in the INT cohort.

*Abbreviations*: CT: chemotherapy; dCCA: distal cholangiocarcinoma; H_MSI: high microsatellite instability; iCCA: intrahepatic cholangiocarcinoma; IHC: immunohistochemistry; L_MSI: low microsatellite instability; MGMT: O6-methylguanine DNA methyltransferase; NGS: next generation sequencing; pCCA: perihilar cholangiocarcinoma.


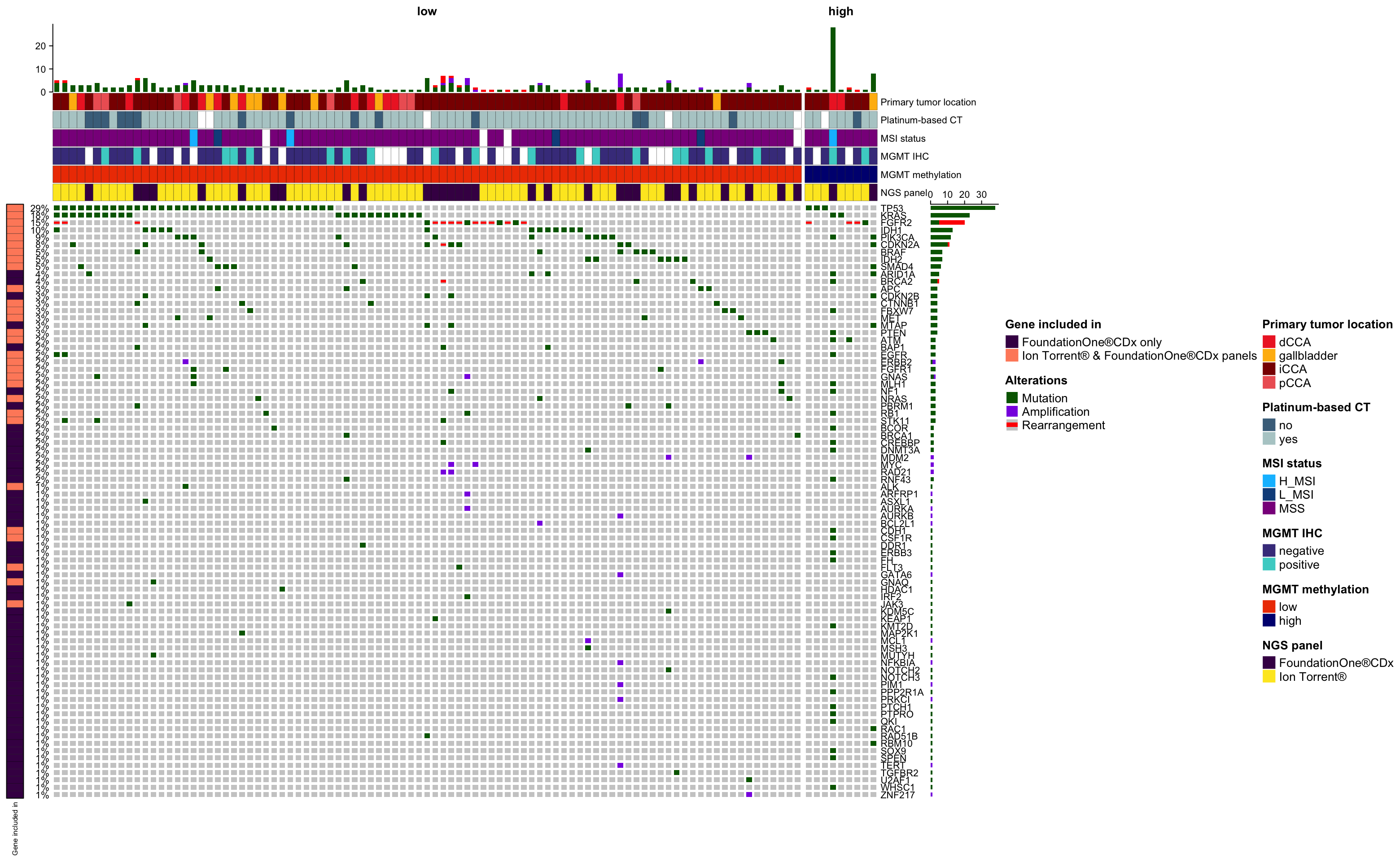


**Supplementary Figure 9.** MGMT mRNA expression (TPM values) according to MGMT promoter methylation in the MASTER BTC cohort.

*Abbreviations*: MGMT: O6-methylguanine DNA methyltransferase; TPM: transcripts per million.


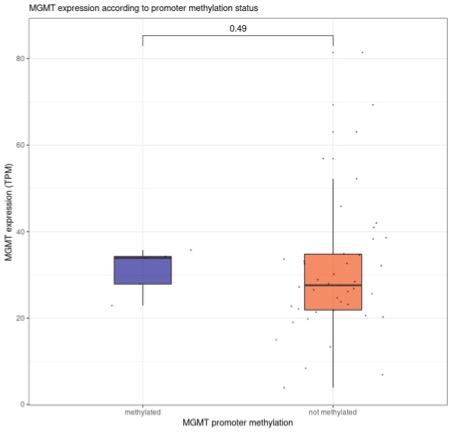


**Supplementary Figure 10**. Swimmer plot of patients treated with temozolomide-based regimens. MGMT promoter methylation values are reported right to each patients’ bar.

Abbreviations: MGMT: O6-methylguanine DNA methyltransferase; TEMIRI: temozolomide plus irinotecan.
